# First Electrochemical Sensor (Screen-Printed Carbon Electrode Modified with Carboxyl Functionalized Multiwalled Carbon Nanotubes) for Ultratrace Determination of Diclofenac

**DOI:** 10.3390/ma13030781

**Published:** 2020-02-08

**Authors:** Agnieszka Sasal, Katarzyna Tyszczuk-Rotko, Magdalena Wójciak, Ireneusz Sowa

**Affiliations:** 1 Faculty of Chemistry, Institute of Chemical Sciences, Maria Curie-Skłodowska University, 20-031 Lublin, Poland; agnieszkaszwagierek@gmail.com; 2 Department of Analytical Chemistry, Medical University of Lublin, 20-093 Lublin, Poland; ireneusz.sowa@umlub.pl

**Keywords:** screen-printed carbon electrode modified with carboxyl functionalized multiwalled carbon nanotubes, diclofenac, differential-pulse adsorptive stripping voltammetry, environmental water samples, HPLC with photo-diode array detection

## Abstract

A simple, sensitive and time-saving differential-pulse adsorptive stripping voltammetric (DPAdSV) procedure using a screen-printed carbon electrode modified with carboxyl functionalized multiwalled carbon nanotubes (SPCE/MWCNTs-COOH) for the determination of diclofenac (DF) is presented. The sensor was characterized using optical profilometry, SEM, and cyclic voltammetry (CV). The use of carboxyl functionalized MWCNTs as a SPCE modifier improved the electron transfer process and the active surface area of sensor. Under optimum conditions, very sensitive results were obtained with a linear range of 0.1–10.0 nmol L^−1^ and a limit of detection value of 0.028 nmol L^−1^. The SPCE/MWCNTs-COOH also exhibited satisfactory repeatability, reproducibility, and selectivity towards potential interferences. Moreover, for the first time, the electrochemical sensor allows determining the real concentrations of DF in environmental water samples without sample pretreatment steps.

## 1. Introduction

Diclofenac (DF) is a nonsteroidal anti-inflammatory drug (NSAID), a derivative of aminophenylacetic acid with a strong anti-inflammatory, analgesic and antipyretic effect. The action of the drug causes the inhibition of inductive (COX-2) cyclooxygenases, responsible for the synthesis of proinflammatory prostaglandins in the site of inflammation and constitutive (COX-1) and the synthesis of prostaglandins fulfilling a physiological function in the gastrointestinal tract and kidneys. DF is absorbed quickly and completely from the gastrointestinal tract and then eliminated completely within 12 h, approximately 60% in the urine and 33% in the faeces. DF is used as an analgesic and anti-inflammatory drug in rheumatoid arthritis, other connective tissue systemic diseases, gout attack, osteoarthritis, and the prevention and treatment of postoperative pain and neuralgia. DF is not recommended for children under 12 years of age and for people suffering from gastric and duodenal ulcer, aspirin-induced asthma, impaired hepatic function, renal insufficiency, and porphyria [1].

In autumn and winter seasons, an increased number of colds and flu is observed, which results in a significant increase in the number of sales and the consumption of pharmaceutical preparations, in particular NSAIDs. Residues of drugs and dietary supplements get into the environment primarily to ground and surface water, causing their pollution. Low water temperatures and short days hinder the process of photolysis and the biodegradation of pharmaceutical preparations, which can cause adverse and unpredictable effects in the ecosystem. The main sources of pollution are factories and production plants, as well as hospitals, health centers, and practically every household. DF is one of the most commonly found ingredients in water, and its concentration is from 3.7 × 10^−11^ to 1.4 × 10^−8^ mol L^−1^ [2,3]. 

Although the likelihood of any form of short-term human health risk after DF release into the environment is low, a study links the catastrophic decline of *Gyps* vulture populations across the Indian subcontinent to DF [4,5]. Generally, NSAIDs such as DF and ibuprofen, for example, have Log Kow values greater than three and may have the capacity to bioaccumulate in the tissues of organisms [6]. DF exhibits acute hepatotoxicity, causes changes in kidneys and gills in rainbow trout (*O. mykiss*) and exhibits acute toxicity to phytoplankton and zooplankton. Moreover, the possibility of synergetic effects with other pharmaceuticals or chemicals in the aquatic environment increases the environmental risk as well [7]. 

There are many methods in the literature that allow for the determination of DF at various concentration levels. The most popular methods are spectrophotometry (determined DF concentrations: 6.8 × 10^−7^–8.4 × 10^−2^ mol L^−1^), spectrofluorimetry (determined DF concentrations: 4.2 × 10^−7^–1.7 × 10^−4^ mol L^−1^), calorimetry (determined DF concentrations: 4.6 × 10^−6^–2.7 × 10^−4^ mol L^−1^), high-performance thin-layer chromatography (determined DF concentrations: 6.8 × 10^−7^–2.7 × 10^−6^ mol L^−1^), and HPLC (determined DF concentrations: 1.7 × 10^−8^–1.4 × 10^−5^ mol L^−1^) [8,9]. Unfortunately, these methods require frequently a time-consuming initial sample preparation stage due to very low concentrations of DF in water samples. 

An alternative to the methods described above are electrochemical methods that allow for quick and cheap analysis of real samples. In the research, various types of working electrodes are used, the most popular electrodes are glassy carbon electrodes modified with metal [10], graphene and carbon nanomaterials [1,11,12,13,14] or organic compounds [15,16,17,18]. In addition, paste electrodes [19] modified with carbon nanomaterials [20,21,22,23,24], silica [25] or organic compounds [26], graphite electrodes [27,28], composite electrodes [27,29], carbon ceramic electrodes [30], boron-doped diamond electrodes [31], and platinum disk electrodes [32,33] are used. The methods presented in most cases are applicable in the determination of DF in pharmaceutical preparations and human urine samples and blood samples. According to our knowledge, currently, there are only four papers on the development of electrochemical sensors for the determination of DF in water samples [15,16,19,31]. However, the authors of those papers determined DF in spiked water samples at concentrations (around 10^−7^–10^−3^ mol L^−1^) higher than those actually present in environmental samples. This was related to the received limit of detection (LOD) values in a range of 3.0 × 10^−8^–2.0 × 10^−7^ mol L^−1^. 

Furthermore, all of the above works describe research methods that are applicable in laboratory analyses. However, no methods have been developed for field analysis. For this purpose, screen-printed sensors can be used. Screen-printing technology allows obtaining a small size of screen-printed sensors, which are characterized by low production cost and high repeatability and allow for the analysis of organic and inorganic compounds at low concentration levels. Screen-printed electrodes have been used in quality control in environmental, clinical, food, and agricultural areas [34]. However, according to the best of our knowledge, there are no papers in the literature that report on the application of screen-printed sensors for the determination of DF.

Due to the huge scale of consumption of pharmaceutical preparations, including DF and their negative impact on the environment and consequently on human health, it is necessary to develop new comprehensive methods for their analytical determination in environmental samples directly in the laboratory field. The aim of this work was to present a new voltammetric procedure for the determination of ultratrace concentrations of DF with a screen-printed sensor. 

## 2. Materials and Methods 

### 2.1. Apparatus

Voltammetric measurements were performed using a µAUTOLAB analyzer (Eco Chemie, Utrecht, The Netherlands) controlled by GPES 4.9 software in a 10 mL electrochemical cell with a commercially available screen-printed sensor (screen-printed carbon electrode modified with carboxyl functionalized multiwalled carbon nanotubes (SPCE/MWCNTs-COOH); DropSens, Llanera, Spain; Ref. 110CNT). This three-electrode system consisted of a working electrode (screen-printed carbon electrode covered by carboxyl functionalized multiwalled carbon nanotubes with a diameter of 4 mm), an auxiliary electrode (SPCE), and a reference electrode (screen-printed silver electrode). In order to characterize the SPCE/MWCNTs-COOH, a commercially available screen-printed carbon electrode (SPCE, DropSens; reference number: C110) was used. The optical profiles and the microscopic images of sensors were recorded using the Contour GT-K1 optical profilometer (Veeco, New York, USA) and a high-resolution scanning electron microscope Quanta 3D FEG (FEI, Hillsboro , USA). Chromatographic analyses were performed on a VWR Hitachi Elite LaChrom HPLC system equipped with a spectrophotometric detector (PDA) and EZChrom Elite software (version 3.3.2 SP2, Merck, Darmstadt, Germany).

### 2.2. Reagents

2-[(2,6-dichlorophenyl)amino]benzeneacetic acid sodium salt (DF, Sigma-Aldrich, Saint Louis, USA) was dissolved in deionized water to prepare a 0.01 mol L^−1^ stock solution of DF. This solution was diluted as required in individual experiments using deionized water. The effects of the type and the pH of the supporting electrolyte on the DF signal were examined using 0.1 mol L^−1^ solutions of H_2_SO_4_, CH_3_COOH, CH_3_COONa + CH_3_COOH (NaAc–HAc) with pH values of 3.5 ± 0.1, 4.0 ± 0.1, 4.2 ± 0.1, 4.5 ± 0.1, 5.0 ± 0.1 and 5.6 ± 0.1, K_2_HPO_4_ + KH_2_PO_4_ with a pH value of 7.0 ± 0.1, (NH_4_)_2_SO_4_ + NH_4_OH with a pH value of 8.3 ± 0.1, NH_4_Cl + NH_4_OH with a pH value of 10.0 ± 0.1, and NaOH with a pH value of 13.0 ± 0.1 prepared from Sigma-Aldrich reagents. Interferences were tested with the use of standard solutions of Ni^2+^, Fe^3+^, Zn^2+^, Pb^2+^, Sb^3+^, Cu^2+^, Cd^2+^, V^5+^, and Mo^6+^ (Merck). The influences of organic substances were investigated based on a reagent obtained from Sigma-Aldrich (ibuprofen, caffeine, paracetamol, and humic acid) and Fluka (Triton X-100, sodium dodecyl sulphate (SDS), and cetyltrimethylamonnium bromide (CTAB)). HPLC-grade acetonitrile and trifluoroacetic acids (TFA) were purchased from Merck (Darmstadt, Germany). The solutions were prepared using ultrapurified water (>18 MΩ cm, Milli-Q system, Millipore, UK).

### 2.3. DF Voltammetric Analysis

Differential-pulse adsorptive stripping voltammetric (DPAdSV) measurements of DF under optimized conditions were carried out in a 0.1 mol L^−1^ NaAc–HAc buffer solution with a pH value of 4.0 ± 0.1. An accumulation potential (*E_acc_*) of −0.25 V was applied when stirring the solution for a period of 60 s (accumulation time was represented by *t_acc_*). After an equilibrium time of 5 s, DPAdSV curves were recorded from −0.25 to 1.5 V with an amplitude (*A*) of 125 mV, a modulation time (*t_m_*) of 10 ms, and a scan rate (*ν*) of 175 mV s^−1^. Then, the background curve was subtracted, and the DPAdSV curves were cut in the potential range of 0.4–0.8 V. The DPAdSV curves for each concentration of DF were recorded 3 times, and the average values of peak currents are shown with an SD for n = 3.

### 2.4. DF Chromatographic Analysis

Chromatographic conditions were based on the literature [35] with a slight modification of the eluent composition. The chromatographic system was as follows: an XB-C18 reversed-phase core-shell column (Kinetex, Phenomenex, Aschaffenburg, Germany) (25 cm of length × 4.6 mm of column diameter, particle size: 5 μm) and a mixture of acetonitrile and water with 0.025% of trifluoroacetic acid (*v*/*v*: 6:4). The flow rate of mobile phase was 1.0 mL min^−1^, and the temperature of thermostat was set at 25 °C. Injection volumes were 20 and 80 µL. All samples were analyzed in triplicate at a wavelength of 276 nm.

### 2.5. Real Sample Application

The voltammetric and chromatographic methods were applied for the determination of DF in Vistula river (Poland) samples stored in sterile, polypropylene containers (Merck), which were collected from two places: at the sewage outlet (sample #1) and about 5 kilometres from the outflow of sewage (sample #2). The water samples were filtered using a 0.45 µm Millipore membrane and then directly analyzed without performing any special sample pretreatment procedure.

## 3. Results and Discussion

### 3.1. Characteristics of SPCE/MWCNTs-COOH Sensors

**Figure 1:**
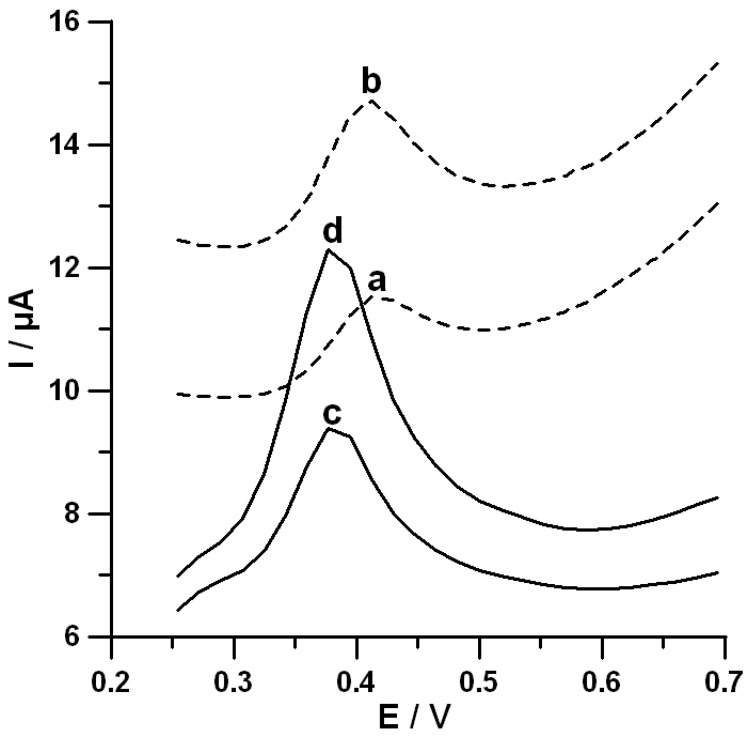
Differential-pulse adsorptive stripping voltammetric (DPAdSV) curves of diclofenac (DF) with different concentrations in a 0.1 mol L^−1^ NaAc–HAc solution with a pH value of 5.0 ± 0.1 at the surface of a bare screen-printed carbon electrode (SPCE) (a,b) and the surface of a screen-printed carbon electrode modified with multiwalled carbon nanotubes (SPCE/MWCNTs-COOH) (c,d). (a) and (c) are for the DF concentration of 0.05 µmol L^−1^. (b) and (d) are for the DF concentration of 0.1 µmol L^−1^. The DPAdSV parameters: accumulation potential (*E_acc_*) of −0.5 V, accumulation time (*t_acc_*) of 30 s, amplitude *(A*) of 100 mV, modulation time (*t_m_*) of 40 ms, and scan rate (*ν*) of 175 mV s^−1^.

In order to understand the use of SPCE/MWCNTs-COOH for the assay of DF, the DPAdSV curves in the DF concentration range of 0.5–200.0 nmol L^−1^ were recorded in a 0.1 mol L^−1^ NaAc–HAc solution with a pH value of 5.0 ± 0.1 at the surface of a bare SPCE and the surface of an SPCE/MWCNTs-COOH. DF showed an oxidation peak at 412 mV with the bare SPCE. At the SPCE/MWCNTs-COOH, the oxidation peak appeared at 377 mV with considerable enhancement in peak current (Figure 1). The oxidation current responses were found to be proportional to the DF concentrations over the ranges of 1.0–200.0 nmol L^−1^ (correlation coefficient r = 0.9997) and 0.5–200.0 nmol L^−1^ (r = 0.9971) at the surfaces of the bare SPCE and the SPCE/MWCNTs-COOH, respectively. The sensitivity values of DF determination at the surfaces of the bare SPCE and the SPCE/MWCNTs-COOH are equal to 0.019 and 0.040 µA/nmol L^−1^, respectively. Compared with the SPCE, the commercially available SPCE/MWCNTs-COOH provided a higher sensitivity and a wider linear range, which was connected with the developed active surface of the sensor modified with carboxyl functionalized carbon nanotubes.

**Figure 2:**
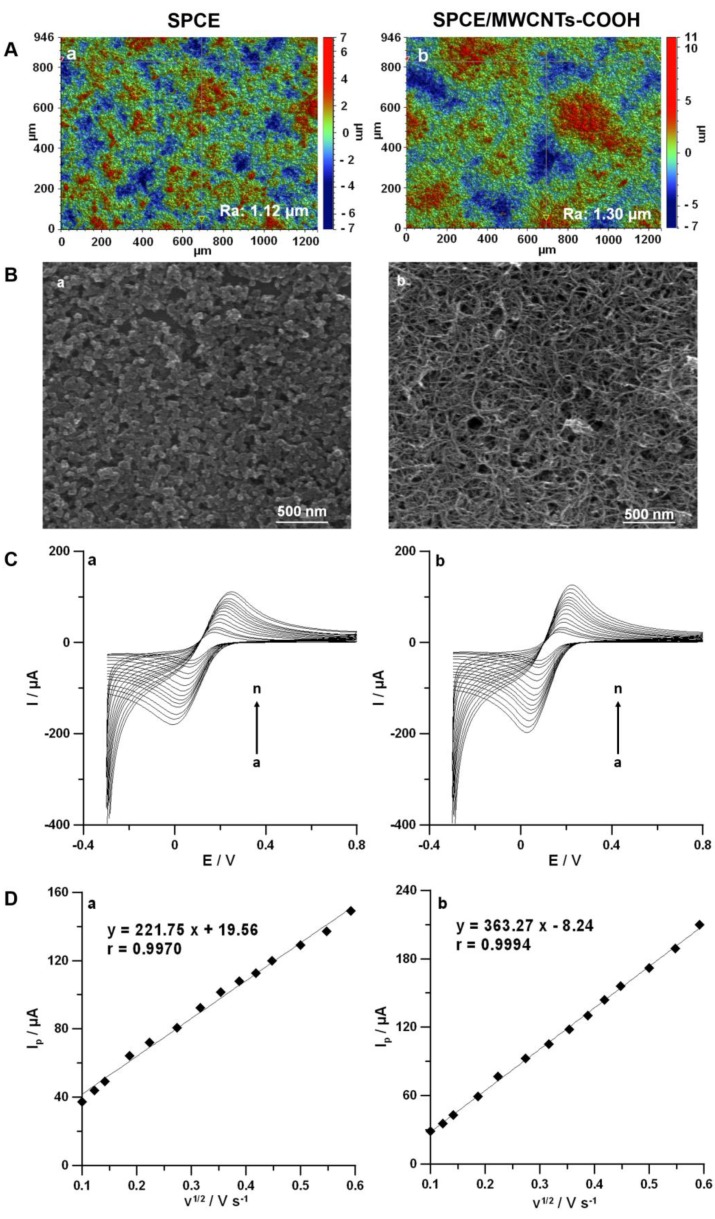
(**A**) Optical profiles. (**B**) SEM images of the SPCE (a) and the SPCE/MWCNTs-COOH (b). (**C**) CV curves recorded at the surfaces of the SPCE (a) and the SPCE/MWCNTs-COOH (b) in a solution of 0.1 mol L^−1^ KCl containing 5.0 mmol L^−1^ K_3_[Fe(CN)_6_] at a *ν* range of 5–500 mV s^−1^. (**D**) Dependences between *I_p_* and *v*^1/2^ for the SPCE (a) and the SPCE/MWCNTs (b).

The morphological studies of SPCE and SPCE/MWCNTs-COOH surfaces were performed using an optical profilometer and a scanning electron microscope. As illustrated in Figure 2A, the modification of the SPCE surface with carboxyl functionalized MWCNTs had a slight impact on the increase of surface roughness (R_a_: 1.12 and 1.30 µm for the SPCE and the SPCE/MWCNTs-COOH, respectively). This confirmed that the whole SPCE surface was covered with a thin layer of carboxyl functionalized MWCNTs, which is also visible in the SEM images (Figure 2B). The carboxyl functionalized MWCNTs were dispersed onto the SPCE without aggregation with special three-dimensional structures and smooth surface [11].

The electrochemical properties of the SPCE and the SPCE/MWCNTs-COOH were also tested using cyclic voltammetry (CV) in a solution of 0.1 mol L^−1^ KCl and 5.0 mmol L^−1^ K_3_[Fe(CN)_6_]. Figure 2C illustrates the CV curves recorded with both electrodes at scan rates of 5–500 mV s^−1^, and Figure 2D shows the dependences between the anodic peak current (*I_p_*) and the square root of scan rate (*v*^1/2^). Based on these results, peak-to-peak separations (*ΔE*) and relative peak separations (*χ^0^)* for a scan rate of 175 mV s^−1^ and active surface areas (*A_s_*) based on the entire range of scan rates were calculated [36]. The results were summarized in Table 1 and indicated the improvement of the reversibility process and electron transfer kinetics by the modification of the SPCE surface with carboxyl functionalized MWCNTs. Moreover, the covering surface with carboxyl functionalized MWCNTs increased the number of active centers, which translated to the enhancement in DF peak current. 

The above described results confirmed the benefits of using the SPCE/MWCNTs-COOH. Therefore, in further experiments, this kind of modified sensor was used for the analysis of DF. 

### 3.2. Optimization of Measurements Solution Composition 

**Figure 3:**
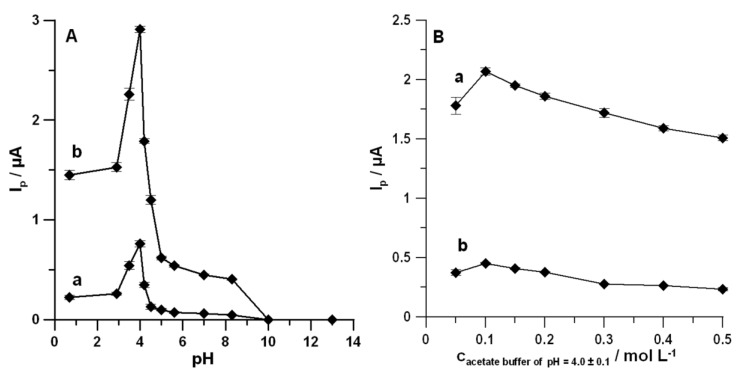
Effects of pH value (**A**) and concentration of the NaAc–HAc buffer solution with a pH value of 4.0 ± 0.1 (**B**) on DF current response. (a) and (b) in (**A**) are for the DF concentration of 0.05 and 0.1 µmol L^−1^, respectively. (a) and (b) in (**B**) are for the DF concentration of 0.05 and 0.1 µmol L^−1^, respectively. Other parameters are the same as in Figure 1.

The effects of the type and the pH of the supporting electrolyte on the voltammetric responses of 0.05 and 0.1 µmol L^−1^ DF were examined using a 0.1 mol L^−1^ solution of H_2_SO_4_, CH_3_COOH and CH_3_COONa + CH_3_COOH (NaAc–HAc) with pH values of 3.5 ± 0.1, 4.0 ± 0.1, 4.2 ± 0.1, 4.5 ± 0.1, 5.0 ± 0.1, and 5.6 ± 0.1, K_2_HPO_4_ + KH_2_PO_4_ with a pH value of 7.0 ± 0.1, (NH_4_)_2_SO_4_ + NH_4_OH with a pH value 8.3 ± 0.1, NH_4_Cl + NH_4_OH with a pH value of 10.0 ± 0.1, and NaOH with a pH value of 13.0 ± 0.1; the corresponding data are listed in Figure 3A. The value of the oxidation peak current of DF increased with the increase of pH value of the solution up to 4.0 ± 0.1 for both studied concentrations of DF. There was no electrochemical response of DF in alkaline media at pH ≥ 10.0 ± 0.1 lacking the presence of enough protons. It was connected with two species of DF (neutral and anionic species), of which the existence depended on pH values. The literature reports that DF presents a pK_a_ of 4.15, so for pH smaller than 4.15 the neutral species predominates, whereas at a greater pH the anionic species predominates [37]. Considering the obtained data, the NaAc–HAc buffer solution with a pH value of 4.0 ± 0.1 is most suitable for the DF determination. 

Furthermore, its concentration was evaluated from 0.05 to 0.5 mol L^−1^ (Figure 3B). The highest values of DF (0.05 and 0.1 µmol L^−1^) signals were attained at a 0.1 mol L^−1^ concentration of the NaAc–HAc buffer solution with a pH value of 4.0 ± 0.1; hence, it was adopted for subsequent experiments.

### 3.3. CV Behaviors of DF with the SPCE/MWCNTs-COOH

**Figure 4:**
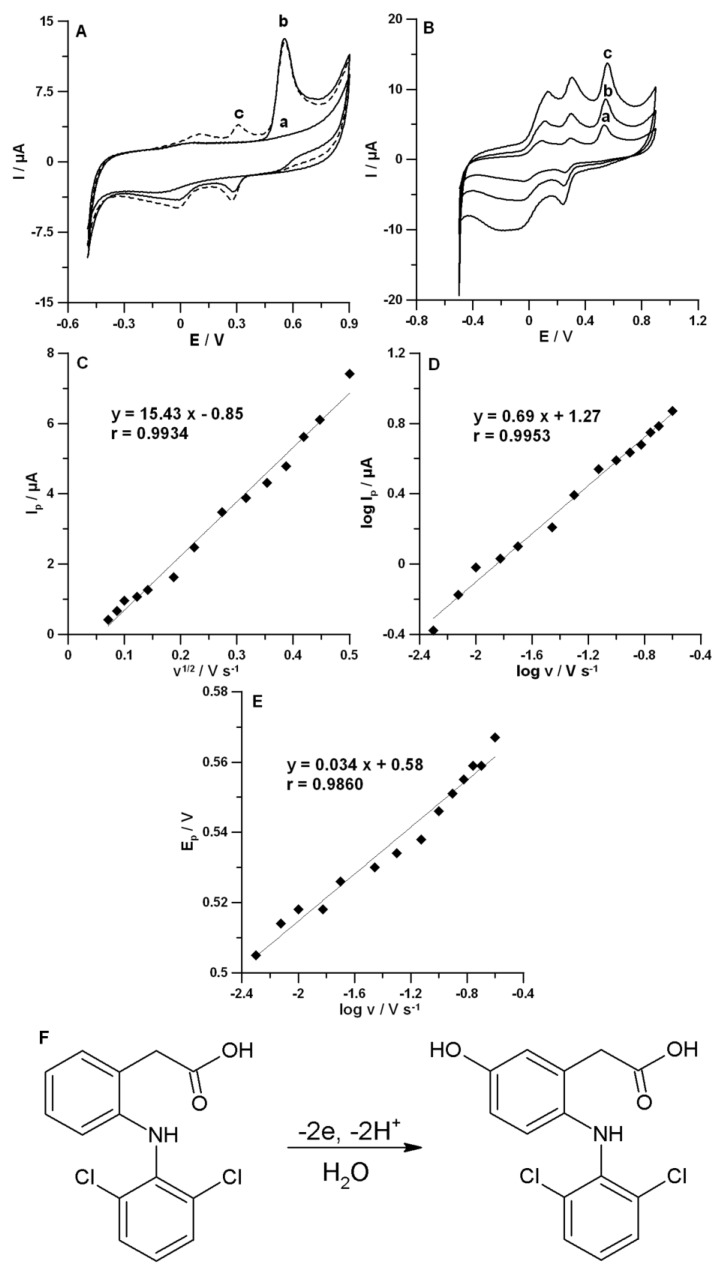
(**A**) CV curves recorded in the 0.1 mol L^−1^ NaAc–HAc buffer solution with a pH value of 4.0 ± 0.1 at *v* equal to 175 mV s^−1^. Curves (a–c) represent CV curves in the solution without DF and with 1.0 µmol L^−1^ DF for the first cycle and the second cycle, respectively. (**B**) CV curves recorded in the 0.1 mol L^−1^ NaAc–HAc buffer solution with a pH value of 4.0 ± 0.1 containing 1.0 µmol L^−1^ DF at different *v* values. Curves (a–c) represent CV curves at *v* equal to 50, 100, and 175 mV s^−1^, respectively. The dependences between *Ip* and *v*^1/2^ (**C**), log*Ip* and log*v* (**D**), and *E_p_* and log*v* (**E**) for *v* from 5 to 250 mV s^−1^. (**F**) Oxidation mechanism of DF

CV curves (first and second cycles) were recorded in the 0.1 mol L^−1^ NaAc–HAc buffer solution with a pH value of 4.0 ± 0.1 containing 1.0 µmol L^−1^ DF using *v* equal to 175 mV s^−1^ (Figure 4A). DF was irreversibly oxidized, giving rise to an oxidation peak at a potential of 555 mV, when the sweep was initiated in the positive direction. In the reverse sweep, two cathodic peaks at potential values of −10 and 275 mV were visible, which formed reversible couples with anodic peaks (102 and 307 mV) observed in the second cycle towards positive potentials. The reversible couples can be created at less positive potentials due to the formation of electrochemically active oxidation products of DF [11,28]. In contrast to the work of Yang et al. [17], the reduction of effective reaction sites at the surface of the SPCE/MWCNTs-COOH by the adsorption of the reaction products of DF was not observed. The oxidation peak currents of DF in the first and second cycles were similar (curves b and c in Figure 4A). Therefore, the regeneration of electrode surface in an additional solution was not required [11]. Further research focused on the DF oxidation peak at a potential of 555 mV with the highest current, which was characterized by the linear increase in the peak current as the concentration increased. 

Valuable information with regard to the nature and the oxidation mechanism of DF at the SPCE/MWCNTs-COOH surface could be obtained by recording CV curves at different values of scan rates. Therefore, the electrochemical behaviors of 1.0 µmol L^−1^ DF in the 0.1 mol L^−1^ NaAc–HAc buffer solution with a pH value of 4.0 ± 0.1 for *v* equal to 5–250 mV s^−1^ were observed. Figure 4B shows the CV curves for the selected *v* of 50, 100, and 175 mV s^−1^. The oxidation peak potential shifted toward more positive values with the increase of scan rate, which confirmed that DF was irreversibly oxidized. The linear relationship between the DF peak current (*Ip*) and the square root of scan rate (*v*^1/2^) (Figure 4C, r = 0.9934) indicated that the oxidation process of DF was controlled by diffusion at the surface of the SPCE/MWCNTs-COOH. However, the slope of 0.69 observed in the plot of log*Ip* vs. log*v* (Figure 4D) indicated that this process was not purely diffusion- or adsorption-controlled [38]. Moreover, with the slope of the peak potential (*Ep*) vs. log(*v*) plot (Figure 4E) using the Laviron's equation [39], the number of electrons involved in the DF oxidation process was calculated. The value determined was equal to 1.51, which proved that two electrons were involved in this process. These results are consistent with the literature data [28,40]. Madsen et al. [40] and Gayal et al. [28] proposed that DF is oxidized to 5-hydrohydiclofenac by losses of 2e^−^ and 2H^+^ according to the overall scheme (Figure 4F). 

### 3.4. Optimization of DPAdSV Parameters 

In order to find the optimum conditions for the DF determination at the surface of the SPCE/MWCNTs-COOH, the influences of various DPAdSV parameters including accumulation potential (*E_acc_*), accumulation time (*t_acc_*), amplitude (*A*), modulation time (*t_m_*), and scan rate (*ν*) on the DF oxidation peak current (0.01 and 0.05 µmol L^−1^) were studied. 

**Figure 5:**
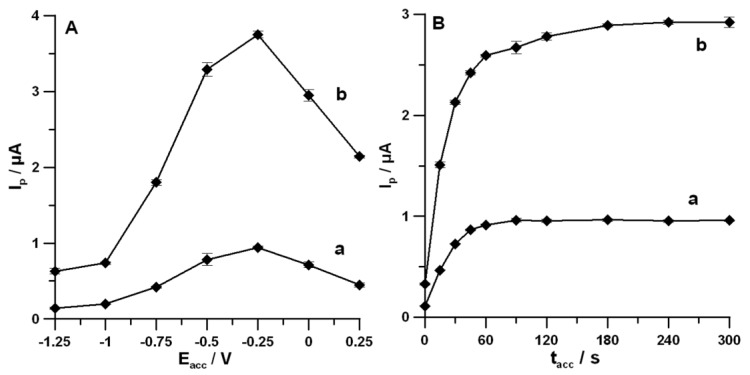
Effects of *E_acc_* (**A**) and *t_acc_* (**B**) on DF current response. (a,b) in (**A**) represent the responses for DF concentrations of 0.01 and 0.05 µmol L^−1^, respectively. (a,b) in (**B**) represent the responses for DF concentrations of 0.01 and 0.05 µmol L^−1^, respectively. The DPAdSV parameters in (**A**) are t_acc_ of 30 s, *A* of 100 mV, *t_m_* of 40 ms, and *ν* of 175 mV s^−1^; the DPAdSV parameters in (**B**) are *E_acc_* of −0.25 V, *A* of 100 mV, *t_m_* of 40 ms, and *ν* of 175 mV s^−1^.

The effects of *E_acc_* and *t_acc_* were tested, because the oxidation process of DF at the SPCE/MWCNTs-COOH surface was not purely diffusion- or adsorption-controlled. The *E_acc_* was varied in the range of −1.25–0.25 V, and *t_acc_* was equal to 30 s. Figure 5A shows that for both studied concentrations of DF, the maximum values of peak current were achieved at *E_acc_* of −0.25 V. Then, at this potential value, *t_acc_* was changed from 0 to 300 s. As can be seen in Figure 5B, taking into account the highest peak currents of DF, *t_acc_* of 60 s can be considered as an optimum condition. 

**Figure 6:**
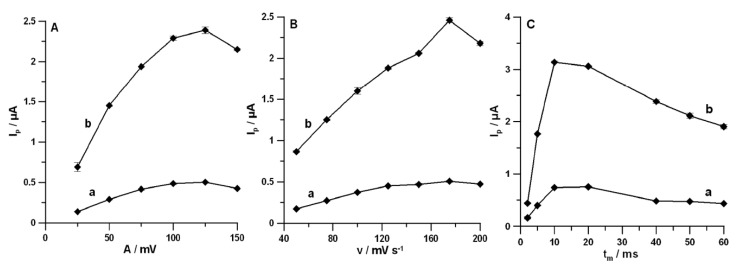
Effects of *A* (**A**), *ν* (**B**), and *t_m_* (**C**) on DF current response. Curves (a,b) are for 0.01 and 0.05 µmol L^−1^ DF, respectively. The DPAdSV parameters: (**A**) *E_acc_* of −0.25 V, *t_acc_* of 60 s, *ν* of 175 mV s^−1^ and *t_m_* of 40 ms; (**B**) A of 125 mV and t_m_ of 40 ms; and (**C**) *A* of 125 mV and *ν* of 175 mV s^−1^.

Moreover, *A* was investigated from 25 to 150 mV (for *ν* of 175 mV s^−1^ and *t_m_* of 40 ms). The highest signals of DF for both studied concentrations were recorded at *A* equal to 125 mV (Figure 6A). Then, the effect of *ν* (50–200 mV s^−1^) on DF peak current at the constant values of *A* (125 mV) and *t_m_* (40 ms) was tested. Figure 6B shows the maximum values of DF peak current at *ν* of 175 mV s^−1^. Furthermore, *t_m_* was varied from 2 to 60 ms (for *A* of 125 mV and *ν* of 175 mV s^−1^). It was found that, for *t_m_* of 10 ms, the highest signals of DF were obtained (Figure 6C). 

### 3.5. Analytical Characteristics

**Figure 7:**
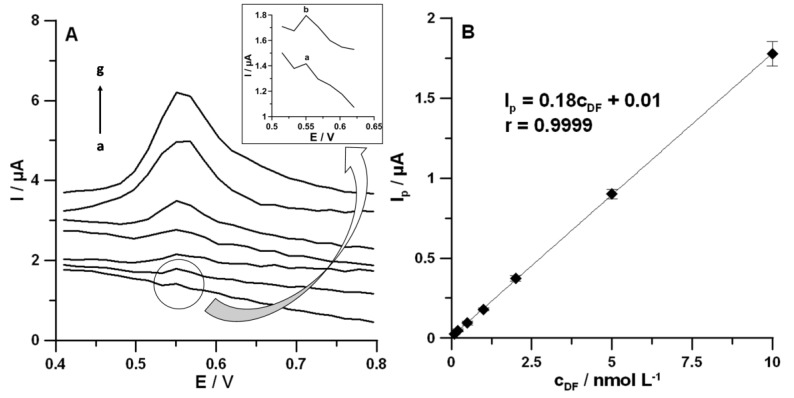
(**A**) DPAdSV curves recorded at the surface of the SPCE/MWCNTs-COOH in the NaAc–HAc buffer solution with a pH value of 4.0 ± 0.1 containing increasing concentrations of DF: (a) 0.1, (b) 0.2, (c) 0.5, (d) 1.0, (e) 2.0, (f) 5.0, and (g) 10.0 nmol L^-1^. (**B**) Calibration graph of DF. The DPAdSV parameters: *E_acc_* of −0.25 V, *t_acc_* of 60 s, *A* of 125 mV, *t_m_* of 10 ms, and *ν* of 175 mV s^−1^.

Under optimized conditions (see the DF voltammetric analysis section), the oxidation current responses were found to be proportional to the DF concentrations over the range of 0.1–10.0 nmol L^−1^. The corresponding results are shown in Figure 7 and Table 2. The sensitivity of DF determination at the SPCE/MWCNTs-COOH was equal to 0.18 ± 0.0070 µA/nmol L^−1^. The LOD and the limit of quantification (LOQ) were 0.028 and 0.094 nmol L^−1^, respectively. The LOD and LOQ values demonstrated that the SPCE/MWCNTs-COOH can be used for analysis of environmental water samples, in which the DF concentration is in the range of 0.037–14.0 nmol L^−1^ [2,3]. It should be clearly emphasized that the SPCE/MWCNTs-COOH allowed obtaining the lowest LOD value compared to all other electrochemical sensors (see Table 3). Moreover, the obtained LOD was significantly lower than those obtained by other techniques applied for DF determination without sample pretreatment steps [8,9]. 

Moreover, the intra-day and inter-day precisions were verified for the determination of 10.0 nmol L^−^^1^ DF with 10 replicates and replicated on five different days, respectively. The results of the intra-day and inter-day precisions were 0.7% and 2.1%, respectively, indicating the satisfactory precious repeatability of the signal at the SPCE/MWCNTs-COOH surface. The reproducibility of the SPCE/MWCNTs-COOH was estimated by recording DPAdSV curves in the solution containing 10.0 nmol L^−^^1^ DF using three commercially available electrodes. The relative standard deviation (RSD) was calculated as 2.9% (n = 9), confirming the acceptable reproducibility of the proposed sensor.

### 3.6. Selectivity of the SPCE/MWCNTs-COOH

In order to confirm the selectivity of the SPCE/MWCNTs-COOH, the DPAdSV responses of DF in the presence of different interferences found in environmental water samples were recorded. The tolerance limit was defined as the concentration, which gave an error of ≤10% in the determination of 10.0 nmol L^−^^1^ DF. It was noticed that ibuprofen (up to 2000-fold excess), caffeine (up to 2000-fold excess), paracetamol (up to 1000-fold excess), Ni^2+^ ions (up to 1000-fold excess), Fe^3+^ ions (up to 1000-fold excess), Zn^2+^ ions (up to 500-fold excess), Pb^2+^ ions (up to 500-fold excess), Sb^3+^ ions (up to 500-fold excess), Cu^2+^ ions (up to 100-fold excess), Cd^2+^ ions (up to 500-fold excess), V^5+^ ions (up to 100-fold excess), and Mo^6+^ ions (up to 100-fold excess) had negligible effects (the relative signals were in the range of 93.7–100.1%) on the assay of DF. Furthermore, the tolerance limits for the studied surfactants and humic acid were 5.0 mg L^−1^ for Triton X-100, 1.0 mg L^−1^ for CTAB, 2.0 mg L^−1^ for SDS, and 10.0 mg L^−^^1^ for humic acid (the relative signals were in the range of 90.4–92.0%). The results confirmed that the proposed procedure offers good selectivity for the determination of DF and the analysis of environmental water samples can by performed without sample preparation.

### 3.7. Application in Environmental Analysis

**Figure 8:**
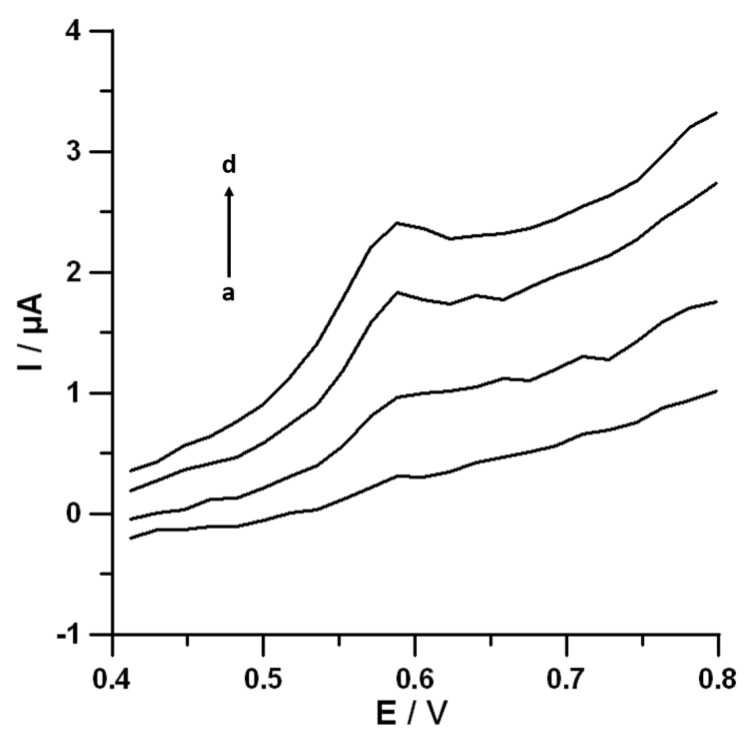
DPAdSV curves recorded at the SPCE/MWCNTs-COOH surface in the course of DF determination in 5 ml Vistula river water sample #1 without DF (a) and with 0.5 nmol L^−1^ (b), 1.0 (c) nmol L^−1^^,^ and 1.5 nmol L^−1^ (d) of DF. Other conditions are the same as in Figure 7.

In order to evaluate the applicability of the voltammetric procedure with the use of SPCE/MWCNTs-COOH for DF determination in environmental water samples, Vistula river water samples collected from two places, at the sewage outlet (sample #1) and about 5 kilometres from the outflow of sewage (sample #2), were analyzed. Table 4 summarizes all results. It should be clearly emphasized that the voltammetric procedure with the use of SPCE/MWCNTs-COOH allowed determining DF at a concentration of 0.42 ± 0.08 nmol L^−1^ in Vistula river water sample #1 without sample pretreatment steps (Figure 8). In sample #2, the DF signal was not visible. The obtained results confirmed the presence of DF in the Vistula river and the dependence of DF concentration on the place of sample collection. 

In order to test the accuracy of the DPAdSV method, samples were spiked with a standard solution of DF. The recovery values attained by the DPAdSV method were between 99.6% and 100.9%, which corresponded to the satisfactory degree of accuracy.

The results with the DPAdSV method were compared with those obtained by the chromatographic method, HPLC/PAD. As can be seen in Table 4, the HPC/PAD method allowed determining DF in river water samples at a concentration of 10.0 nmol L^−1^, which was significantly above the real DF concentration in water samples. In order to determine lower concentrations of DF by the chromatographic method, an additional sample preparation step was required. According to the Student’s t-test, there were no significant differences between DF concentrations obtained by both methods. The calculated t values (*t_exp_*) were 0.48 and 0.60, which were below the critical value equal to 1.81 (for degrees of freedom f equal to 4 (f = n_1_ + n_2_ - 2) and 95% confidence level). All these results indicated that the DPAdSV procedure using an SPCE/MWCNTs-COOH is highly selective and excellent for the determination of DF in real applications. 

## 4. Conclusions

In summary, a simple, sensitive and time-saving DPAdSV procedure using an SPCE/MWCNTs-COOH was presented and successfully applied for the determination of DF. The modified sensor showed an improved sensing activity towards DF compared to a bare SPCE due to the effect of the modifier. The use of carboxyl functionalized MWCNTs improved the electron transfer process and the active surface area of the sensor. The SPCE/MWCNTs-COOH exhibited excellent current responses towards DF determination in the linear range of 0.1–10.0 nmol L^−1^ and an LOD value of 0.028 nmol L^−1^. Furthermore, the SPCE/MWCNTs-COOH also showed satisfactory repeatability, reproducibility, and selectivity towards potential interferences. It should be clearly stressed that, for the first time, the electrochemical sensor was used for the determination of a real concentration of DF (0.42 ± 0.08 nmol L^−1^) in environmental water samples (Vistula river samples) without sample pretreatment steps. All these discussion indicated that the DPAdSV procedure using an SPCE/MWCNTs-COOH has great potential towards the determination of DF in real water samples to maintain environmental protection. Moreover, the DPAdSV procedure using an SPCE/MWCNTs-COOH can be applied in the DF field analysis.

## Figures and Tables

**Table 1:** Electrochemical characteristics of the SPCE and the SPCE/MWCNTs-COOH using cyclic voltammetry (CV) in a solution of 0.1 mol L^−1^ KCl and 5.0 mmol L^−1^ K_3_[Fe(CN)_6_].

Calculated parameter	SPCE	SPCE/MWCNTs-COOH
*ΔE* for *v* of 175 mV s^−1^	189.0 ± 1.9 mV (n = 3)	149.0 ± 1.5 mV (n = 3)
*χ^0^* for *v* of 175 mV s^−1^	3.26 ± 0.031 (n = 3)	2.57 ± 0.025 (n = 3)
*A_s_* for *v* of 5–500 mV s^−1^	0.061 ± 0.00058 cm^2^ (n = 3)	0.10 ± 0.00097 cm^2^ (n = 3)

**Table 2:** Characteristics of the calibration plot of DF obtained at the SPCE/MWCNTs-COOH surface.

Parameter	DPAdSV
Linear range (nmol L^−1^)	0.1–10.0
Accumulation time (s)	60
Slope (b) ± SD_b_ (n = 3) (µA/nmol L^−1^)	0.18 ± 0.0070
Intercept (a) ± SD_a_ (n = 3) (µA)	0.010 ± 0.0017
Correlation coefficient (r)	0.9999
Limit of detection (LOD; nmol L^−1^)	0.028
Limit of quantification (LOQ; nmol L^−1^)	0.094
Intra-day precision (RSD, n = 10) (%)	0.7
Inter-day precision (RSD, n = 15) (%)	2.1
Reproducibility (RSD, n = 9) (%)	2.9

LOD = 3SD_a_/b and LOQ = 3SD_a_/b [41]; RSD: relative standard deviation for a DF concentration of 10.0 nmol L^−1^.

**Table 3:** Comparison of electrochemical methods for the determination of DF.

Electrode	Method	Linear Range (mol L^−1^)	Detection Limit (mol L^−1^)	Application	Ref.
n-GCE	CV	2.0 × 10^−4^–1.5 × 10^−3^	2.8 × 10^−5^	pharmaceutical formulations	[10]
NiNPs/ERGO/GCE	SWV	2.5 × 10^−7^–1.3 × 10^−4^	9.0 × 10^−8^	pharmaceutical formulations, urine samples	[1]
AuNP/MWCNT/GCE	SWV	3.0 × 10^−8^–2.0 × 10^−4^	2.0 × 10^−8^	pharmaceutical formulations, urine samples	[11]
MWCNTs/ Cu(OH)_2_/IL/GCE	DPV	1.8 × 10^−7^–1.2 × 10^−4^	4.0 × 10^−8^	pharmaceutical formulations	[12]
MWCNT-IL/CCE	DPV	5.0 × 10^−8^–2.0 × 10^−5^	2.7 × 10^−8^	blood plasma samples	[13]
GO-COOH/GCE	LSV	1.2 × 10^−6^–4.0 × 10^−4^	9.0 × 10^−8^	urine samples, blood serum samples	[14]
GCE/Amino-AT	SWV	3.0 × 10^−7^–2.0 × 10^−5^	2.0 × 10^−7^	pharmaceutical formulations, spiked water samples	[15]
GCE/APTES-Amino-AT-Silica			5.3 × 10^−8^		
PDDA-GR/GCE	DPV	1.0 × 10^−5^–1.0 × 10^−4^	6.1 × 10^−7^	pharmaceutical formulations, spiked lake water samples	[16]
MWNTs–DHP/GCE	CV	1.7 × 10^−7^–2.5 × 10^−6^ 2.5 × 10^−6^–7.5 × 10^−5^	8.0 × 10^−8^	pharmaceutical formulations	[17]
DBA/GCE	CV	1.0 × 10^−5^–1.0 × 10^−3^	2.7 × 10^−7^	blood serum samples	[18]
CPE	SWV	1.0 × 10^−6^–1.0 × 10^−5^	2.0 × 10^−7^	spiked model water samples	[19]
MWCNTs/CoHCF/IL/PE	DPV	1.0 × 10^−3^–1.0 × 10^−1^	3.0 × 10^−4^	pharmaceutical formulations, urine samples	[20]
Fe_3_O_4_@SiO_2_/MWCNTs-CPE	SWV	5.0 × 10^−7^–1.0 × 10^−4^	4.0 × 10^−8^	pharmaceutical formulations, blood serum samples	[21]
VFMCNTPE	SWV	2.5 × 10^−6^–6.0 × 10^−4^	2.0 × 10^−6^	pharmaceutical formulations, urine samples	[22]
IL/CNTPE	DPV	5.0 × 10^−7^–3.0 × 10^−4^	2.0 × 10^−7^	pharmaceutical formulations, urine samples	[23]
IL/CNTPE	SWV	3.0 × 10^−7^–7.5 × 10^−4^	9.0 × 10^−8^	pharmaceutical formulations, urine samples	[24]
Silica NPs-CPE	DPV	1.0 × 10^−7^–5.0 × 10^−4^	4.6 × 10^−8^	pharmaceutical formulations	[25]
TCPE	DPV	1.0 × 10^−5^–1.4 × 10^−4^	3.3 × 10^−6^	pharmaceutical formulations, urine samples	[26]
PTFE-G; EG; E-CB	DPV	6.0 × 10^−8^–1.0 × 10^−6^	5.0 × 10^−8^	pharmaceutical formulations	[27]
EPPG	SWV	1.0 × 10^−8^–1.0 × 10^−6^	6.2 × 10^−9^	pharmaceutical formulations, urine samples	[28]
CuZEGE	CV, DPV	2.0 × 10^−5^–3.0 × 10^−7^	5.0 × 10^−8^	-	[29]
MWCNT-IL/CCE	DPV	5.0 × 10^−8^–5.0 × 10^−5^	1.8 × 10^−8^	pharmaceutical formulations, blood plasma samples	[30]
BDDE	DPV	3.1 × 10^−7^–3.1 × 10^−5^	3.0 × 10^−8^	spiked tap water samples	[31]
PtDE	DPV	5.0 × 10^−6^–5.9 × 10^−5^	1.0 × 10^−6^	pharmaceutical formulations, blood serum samples	[32]
PtDE	SWV	5.1 × 10^−6^–5.9 × 10^−5^	1.7 × 10^−6^	pharmaceutical preparations, blood serum samples	[33]
SPCE/MWCNTs-COOH	DPAdSV	1.0 × 10^−10^–1.0 × 10^−8^	2.8 × 10^−11^	river water samples	This work

**Table 4:** Results of DF determination in Vistula river water samples.

Sample	DF concentration ± SD (nmol L^–1^) (n = 3)			Recovery (%)	t_exp_
	Added	Found with the DPAdSV procedure	Found with the HPLC/PAD method	DPAdSV	
#1	0	0.42 ± 0.08	-	-	-
#1	5.0	5.40 ± 0.20	-	99.6	-
#1	50.0	50.80 ± 1.40	52.30 ± 4.08	100.5	0.60
#2	0	-	-	-	-
#2	0.4	0.40 ± 0.01	-	100.0	-
#2	5.0	5.38 ± 0.33	-	99.6	-
#2	50.0	51.0 ± 0.90	49.80 ± 4.25	100.9	0.48
